# Oxidative Stress in Genetic Cataract Formation

**DOI:** 10.3390/antiox13111315

**Published:** 2024-10-29

**Authors:** James Fielding Hejtmancik

**Affiliations:** Ophthalmic Molecular Genetics Section, Ophthalmic Genetics and Visual Function Branch, National Eye Institute, National Institutes of Health, Bethesda, MD 20892, USA; hejtmancikj@nei.nih.gov

**Keywords:** lens, cataract, genetics

## Abstract

Background: Cataracts are the leading cause of blindness worldwide, and age-related cataracts are the result of environmental insults that largely lead to oxidative stress imposed on a genetic background that determines susceptibility to these stresses. Methods: A comprehensive literature review was performed to identify GWAS, targeted association studies, and TWAS that identified genes associated with age-related cataract. Additional genes associated with age-related cataracts were identified through the CAT-MAP online database. Pathway analysis was performed using Qiagen Ingenuity Pathway Analysis and pathways related to oxidative stress were analyzed using the same program. Results: A large number of genes have been identified as causes of both Mendelian and complex cataracts. Of these, 10 genes related to oxidative stress were identified, and all were associated with age-related cataracts. These genes fall into seven canonical pathways primarily related to glutathione metabolism and other pathways related to detoxifying reactive oxygen species. Conclusions: While a relatively small number of antioxidant related genes were identified as being associated with cataracts, they allow the identification of redox pathways important for lens metabolism and homeostasis. These are largely related to glutathione and its metabolism, other pathways for detoxification of reactive oxygen species, and the transcriptional systems that control their expression.

## 1. Introduction

The ocular lens is a transparent cellular structure responsible for transmitting and fine-focusing light rays onto the retina, which, in turn, sends visual impulses to the visual cortex via the optic nerve and visual pathways. The lens accounts for about one-third of the total refractive power of the eye with the clear cornea accounting for most of the rest. Cataracts refer to opacification of the eye lens and can occur in isolation or associated with other ocular and/or multisystem disorders. Other genetic disorders affecting lens formation, size, shape, position in the eye, or embryonic vascular regression are much less common and may present with or without cataracts and/or other ocular or systemic diseases. This review briefly summarizes the molecular genetic etiology of cataracts and delineates the role that oxidative stress and the lens defense systems to resist it have in inherited cataracts.

## 2. Materials and Methods

### 2.1. Literature Review

Pubmed (https://pubmed.ncbi.nlm.nih.gov/38984127/, accessed on 24 September 2024) was searched with the keywords ‘cataract’ and ‘genome’ and ‘association’ and ‘age’ in all combinations and papers describing GWAS of cataracts were identified manually. The CAT-MAP (https://cat-map.wustl.edu/, 1 April 2024 version) database was screened manually for antioxidant-related genes, as were recent reviews covering inheritance of age-related cataracts [1,2]. All genes were shown as being related to age-related cataracts were included. For use in the IPA analysis all genes in CAT-MAP listed as having a complex inheritance or being related to age-related cataracts were included. Gene symbols and abbreviations are listed in Supplementary Table S6.

### 2.2. IPA Analysis

The networks and functional analyses were generated through the use of QIAGEN Ingenuity Pathway Analysis (https://digitalinsights.qiagen.com/products-overview/discovery-insights-portfolio/analysis-and-visualization/qiagen-ipa/, accessed on 1 October 2024) [3] accessed on 20 September 2024. A dataset containing gene identifiers from GWAS and meta-analyses described above [4,5,6,7,8,9,10,11,12,13], as well as genes listed as having complex inheritance or identified as being associated with age-related cataracts in the April, 2024 version of CAT-MAP [14] (https://cat-map.wustl.edu, https://www.omim.org), shown as GWAS + CAT-MAP + TWAS in Table S2, was uploaded into the application. Each identifier was mapped to its corresponding entity in QIAGEN’s Knowledge Base. These molecules, called Network Eligible molecules, were overlaid onto a global molecular network developed from information contained in the QIAGEN Knowledge Base. Networks of Network Eligible Molecules were then algorithmically generated based on their connectivity. Canonical pathways analysis identified the pathways from the QIAGEN Ingenuity Pathway Analysis library of canonical pathways that were most significant to the data set. Molecules from the data set that were associated with a canonical pathway in the QIAGEN Knowledge Base were considered for the analysis. The significance of the association between the data set and the canonical pathway was measured in two ways: (1) A ratio of the number of molecules from the data set that map to the pathway divided by the total number of molecules that map to the canonical pathway is displayed; and (2) A right-tailed Fisher’s Exact Test was used to calculate a p-value determining the probability that the association between the genes in the dataset and the canonical pathway is explained by chance alone. Because the gene list did not include quantitative changes in expression Z-scores could not be calculated for the pathways.

Networks are shown as a graphical representation of the molecular relationships between molecules. The figures are generated using the Path Designer function of IPA. Molecules are represented as nodes, and the biological relationship between two nodes is represented as an edge (line). All edges are supported by at least one reference from the literature, from a textbook, or from canonical information stored in the QIAGEN Knowledge Base. Human, mouse, and rat orthologs of a gene are stored as separate objects in the QIAGEN Knowledge Base but are represented as a single node in the network. Nodes are displayed using various shapes that represent the functional class of the gene product. Edges are displayed with various labels that describe the nature of the relationship between the nodes (e.g., P for phosphorylation, T for transcription).

## 3. Classification of Inherited Cataracts by Their Onset and Clinical Morphology

Light scattering in the lens occurs when the refractive index varies significantly over distances approximating the wavelength of transmitted light [15,16], and when this causes cloudiness sufficient to decrease vision it is termed a cataract. As lens transparency and refraction require both high concentrations of closely packed crystallins in the cytoplasm of lens cells and an appropriate microarchitecture, variations of the refractive index can occur through two processes: destabilization of soluble proteins resulting in protein aggregation of sufficient size to scatter light and/or disruptions of the microarchitecture of the lens fiber cells with cellular disarray and vacuole formation [1]. The age of onset, or in practice the age-at-diagnosis, of a cataract provides a reasonable way to categorize them and relates generally to their etiology. A cataract that is present at birth is termed congenital, one that occurs within the first two years of life infantile, and within the first decade childhood or juvenile. Cataracts occurring between the ages of 15 and 20, and 40 and 50, years of age are termed pre-senile, and those presenting after this are considered age-related or senile. The latter are the most common forms of cataract, accounting for about 40% of visual impairment worldwide [17]. 

Age-related cataract is generally regarded as a multifactorial disease involving complex interactions between environmental and genetic factors [1,18,19], whose incidence increases with age, so that by 80 years of age approximately 68% of the United States population is estimated to have age-related cataract [20]. In contrast, Mendelian inherited cataracts, especially bilateral, most often show an early-onset, often in the first year or years of life [2,21,22,23]. Depending on the level of development of the country, congenital cataracts are estimated to have a prevalence of 4.24/10,000 births and childhood cataracts have been estimated to have a cumulative risk of 108.4/100,000 children [24]. Although environmental causes are more common in developing than economically advanced countries, inherited cataracts contribute approximately 22% of childhood cataracts globally, resulting in an estimated 200,000 children blind from cataracts [25]. In addition to the direct effects of the cataract, irreversible cortical blindness may result from lack of visual stimulation during the sensitive period in infancy [26], although considerable uncertainty remains about the optimum procedures and timing of cataract surgery in this age group [27].

In addition to the age of onset, the clinical classification of cataracts inherited in a Mendelian fashion is most often based on the morphology and location of lens opacities. The most commonly used system is that proposed by Merin, which divides cataracts into four general types: zonular, polar, total (also termed mature), and membranous (also termed capsular) [28]. Zonular cataracts involve a limited area of the lens and can be divided into nuclear (affecting the lens core, often the fetal nucleus), lamellar (affecting growth layers of lens fiber cells around the lens core), and sutural (affecting the Y-sutures). Polar cataracts localize to the anterior or posterior poles of the lens and often also involve the overlying lens capsule. Total and membranous cataracts are often end stages of other cataract types with total cataract often representing progression of a nuclear cataract to include the entire lens and membranous cataracts resulting from rupture or resorption of the lens body followed by fusion of the anterior and posterior capsules. Other cataract descriptions relate to specific cataract shapes (e.g., aculeiform, coraliform, floriform, polymorphic, disc-like), density (e.g., pulverulent or dust like), and color (e.g., blue-dot or cerulean). 

In most studies the three main types of age-related cataract are nuclear, cortical, and posterior sub-capsular, which may occur either alone or in combination. These can show varying degrees of clinical severity, which can be quantitated by a number of metrics including the Oxford clinical cataract classification and grading system [29], The Age Related Eye Disease Studies (AREDS) system [30], or the Lens Opacities Classification System (LOCS)-III system [31]. Both epidemiological and genetic evidence suggests a different, but perhaps overlapping, environmental etiologies for the three main types of age-related cataract [19,32]. Although the specific environmental risk factor associations vary among cataract types, oxidative insult appears to be the final pathway that combines them and is the overriding pathogenic mechanism [33]. This is especially true as the lens ages and metabolic support of the quiescent nuclear fiber cells by the metabolically more active epithelia and cortical fibers is impeded by development of a diffusion barrier for small molecules such as glutathione [34]. While estimates of the heritability of age-related cataracts have varied from 48% for nuclear and 37–58% for cortical cataract [35,36,37,38], the specific genetic etiologies identified through linkage, association, and functional studies account for only a small fraction of this effect.

## 4. Genes and Pathways Implicated in Mendelian and Age-Related Cataracts

Currently at least 59 genes have been identified as causing Mendelian inherited forms of primary cataracts, in addition to more than 200 genetic diseases that have been shown to be associated with secondary forms of cataracts (https://cat-map.wustl.edu, https://www.omim.org) [39]. Interestingly, genome wide association studies (GWAS) suggest that polymorphisms in a number of genes underlying inherited congenital or childhood cataracts may contribute to susceptibility for childhood, presenile, or age-related cataracts [4,5], although the overlap is not complete. Approximately 33% of Mendelian congenital cataracts are caused by mutations in lens crystallins, 26% by mutations in growth factors and their receptors, 18% by mutations in connexins, 11% by mutations in membrane proteins or transporters, 4% by mutations in chaperones or protein degradation apparatus, 4% by mutations in intermediate filament proteins, 1% in other categories or proteins and the remainder are unknown [1]. When the set of Mendelian congenital cataract genes in CAT-MAP is analyzed for pathways in IPA, no pathways related to oxidative damage or antioxidants are identified. Severe mutations in a number of genes, including BFSP2, HSF4, EPHA2, CRYAA, CHMP4B, GJA8, SLC16A12, CRYGD, CRYGS, CRYBA2, CRYBA3, CRYBB2, LIM2, and PITX3 can cause congenital cataracts, while milder mutations cause progressive childhood or presenile cataracts [1]. However, two large multiethnic GWAS meta-analyses [4,5], one in a multiethnic Asian population [6], one in European Americans [7], one in a Scottish diabetic cohort [8], one in a Taiwanese diabetic cohort [9], a mitochondrial study in a Latino population [10], one in a Finnish population [11], one in a Mayan cohort [12], and a transcriptome wide association study (TWAS) study [13] identified only three genes, DNMBP, SOX2, and CRYAA, implicated in congenital cataracts as also contributing to age-related cataracts, out of 157 candidate genes identified (including 87 identified through GWAS), even though the first locus identified for age-related cataracts, EPHA2, can also cause congenital cataracts [40]. The candidate genes identified in each of these studies is summarized in Table S1. 

Thus, while the genetic etiology of age-related cataracts is much less well understood, the groups of genes that are implicated in congenital cataracts appear to account for only a small fraction of their genetic etiology. Table S2 shows lists of the age-related cataract genes identified in genome wide association studies and in a combination of the genome wide transcription association study, with and without genes not identified in GWAS or transcriptomics screens but identified through targeted association studies and included in CAT-MAP. The targeted association studies included in CAT-MAP tend to have much smaller sample sizes, which might increase the risk of false-positive associations, but also tend to be based on a single population, which might uncover associations specific to that population but lost when combining multiple populations with different risk associations. The only crystallin included in the list is CRYAA, and the only well-studied lens transcription factors are MAF and SOX2, while FTL, which can cause Mendelian cataracts, has also been associated with age-related cataracts. A summary of genes related to oxidative stress included in CAT-MAP or one or more age-related cataract GWAS studies is shown in Table 1. While including the transcriptomic analysis roughly doubles the number of genes identified (167 vs. 83 or 243 vs. 172 if CAT-MAP genes are included), there is only a small increase in the number of canonical pathways significantly associated with age-related cataracts (136 vs. 122), and there is a high degree of overlap between the two groups of pathways. This suggests that the genes and thus the pathways included in both congenital and age-related cataracts might be important for lens development, homeostasis, and clarity.

**Figure 1:**
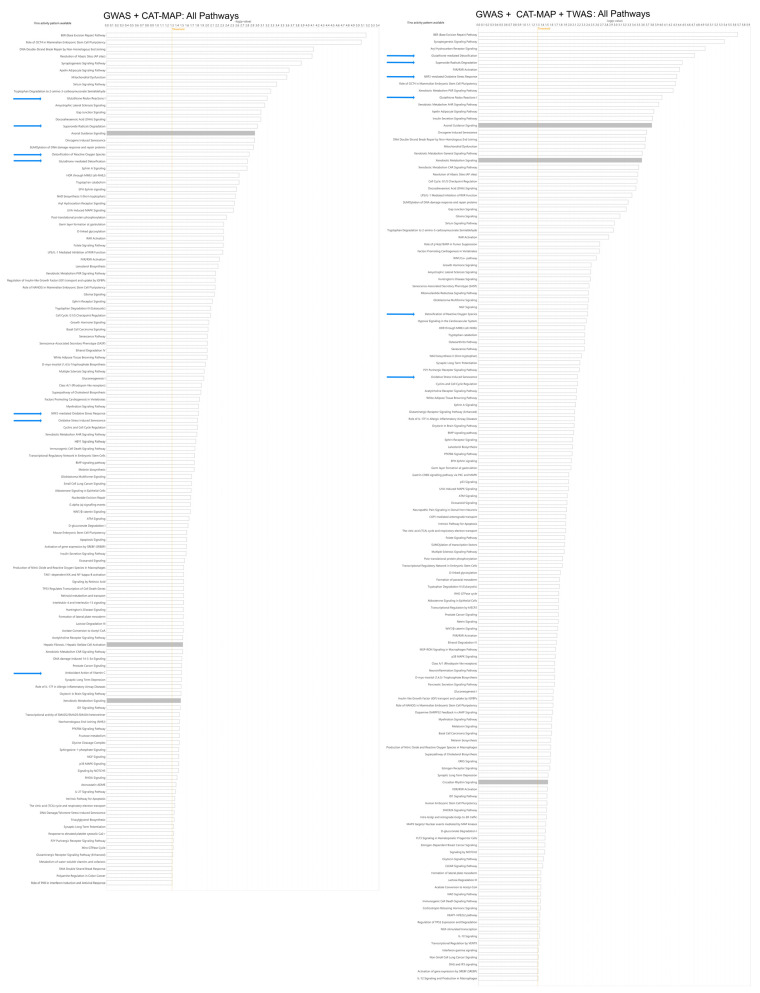
Summary of IPA canonical pathways enriched in genes associated with age-related cataracts. The left column includes genes identified in association studies, either GWAS or targeted studies listed in CAT-MAP. The right column also includes pathways enriched in genes identified through the transcriptome wide association study. Antioxidant related pathways are marked by blue arrows and corrected *p* values are shown as horizontal bars with a significance level of 0.05 shown by an orange line.

While the pathways significantly associated with age-related cataracts are highly diverse, ranging from transcriptional and regulatory pathways (114 of 137 significantly associated pathways) to sumoylation (2 pathways), 7 of them relate to antioxidation, defense against oxidative insults, or regulation of antioxidant factors (Figure 1, Tables S3 and S4). Table S3 shows the pathways enriched in the genes included in the combined GWAS studies and CAT-MAP, while Table S4 includes pathways identified by these genes and the Transcriptome wide association study (TWAS) as well. The GWAS only pathways include seven pathways associated with antioxidation, while the combined GWAS and transcriptomic association includes six. In the GWAS + CAT-MAP group 4 of the top 18 pathways are related to antioxidants, while in the GWAS + CAT-MAP + TWAS 4 if the top 10 pathways are. The top pathways in both of these groups include glutathione redox reactions, glutathione-mediated detoxification, superoxide radical degradation. The NRF2 (NFE2L2) mediated oxidative stress response, and detoxification of reactive oxygen species pathways are found among the top pathways of one group and further down in the other. In addition, both groups include the oxidative stress induced senescence pathway and the GWAS + CAT-MAP only group includes the antioxidant action of vitamin C pathway although these are less strongly associated. As is suggested by the pathway names and the limited number of genes from which the pathways are derived, the included pathways contain considerable overlap. The cataract genes associated with each of the antioxidant pathways are shown in Table S5. 

**Figure 2:**
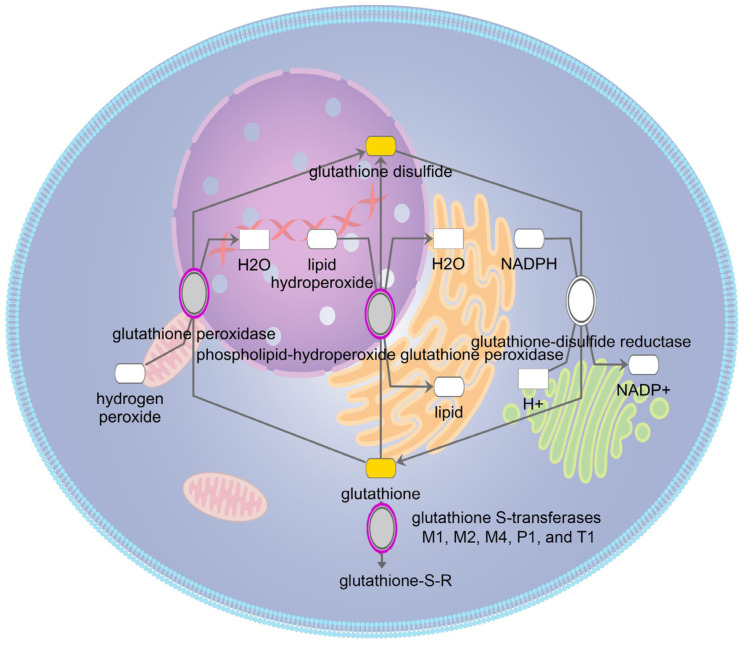
Schematic diagram of glutathione redox reactions. Genes associated with age-related cataracts are shaded gray and glutathione and its metabolites are shaded yellow.

**Figure 3:**
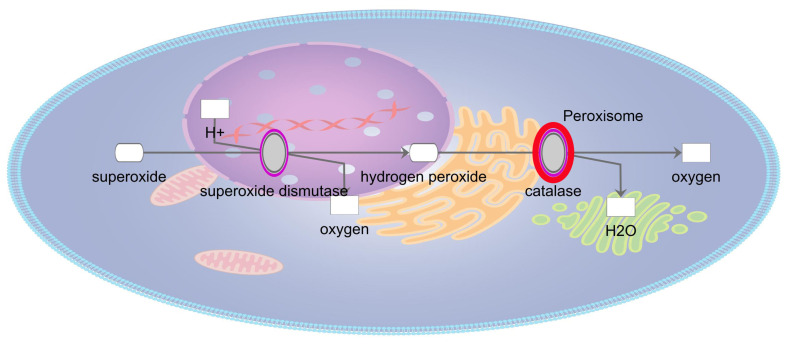
Schematic diagram of the superoxide radical degradation pathway. Genes associated with age-related cataracts are shaded gray.

**Figure 4:**
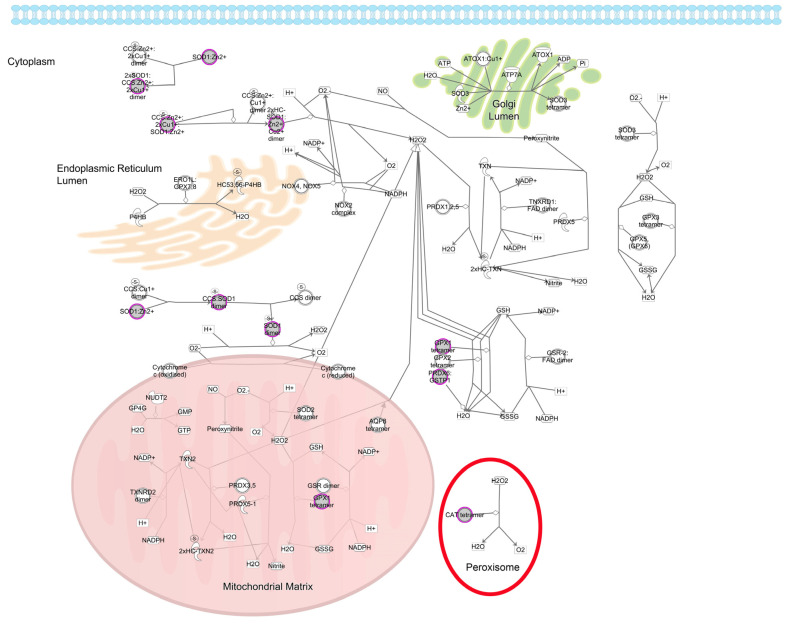
Schematic diagram of the detoxification of reactive oxygen species pathway. Location within the cell is shown for cytoplasmic, nuclear, and peroxisomal proteins. Genes associated with age-related cataracts are shaded gray.

It is informative to look more closely at the redox pathways in which the age-related cataract implicated genes participate. One of the most important reducing pathways in the lens is the glutathione redox cycle [49]. The lens maintains high levels of glutathione to maintain a reduced environment. Similarly, the enzymes of the glutathione redox cycle are distributed throughout the cytoplasm (Figure 2) [50]. While glutathione peroxidase, which detoxifies hydrogen peroxide and lipid hydroperoxides by oxidizing glutathione to glutathione disulfide, has been implicated in age-related cataracts [44], glutathione reductase, which in turn reduces glutathione disulfide to glutathione, has not. Also, glutathione peroxidase 1, the gene implicated in age-related cataracts, has higher activity on hydrogen peroxide than lipid hydroperoxides, suggesting this is the critical oxidant relevant to age related cataracts [51]. In addition to the glutathione redox cycle, the μ, π, and θ families of glutathione S-transferase, which detoxify oxidized substrates by transferring reduced glutathione to them, are also implicated in age-related cataracts [52]. Interestingly, glutathione S-transferases have also been shown to influence apoptotic pathways, which have been shown to be activated in congenital and probably some age-related cataracts [1,53]. In addition to glutathione related pathways the lens uses superoxide dismutase to convert superoxide radicals to oxygen and hydrogen peroxide (Figure 3). The hydrogen peroxide is then either detoxified as above or converted to water and oxygen by catalase (Figure 3) [54]. Both superoxide dismutase and catalase have been implicated in age-related cataracts, emphasizing the importance of this pathway for lens homeostasis, even though levels of superoxide dismutase 1, the cytoplasmic form implicated in age-related cataracts, is relatively low in the lens [55]. In contrast to superoxide dismutase 1 and the proteins in the glutathione redox cycle, catalase is localized to peroxisomes and is most active at higher levels of hydrogen peroxide than is glutathione peroxidase [56,57]. This is consistent with Gpx1 but not Cat knockout mice developing age-related cataracts [58,59]. A summary of the protein products of these genes and the pathways in which they are active is provided in Figure 4. 

**Figure 5:**
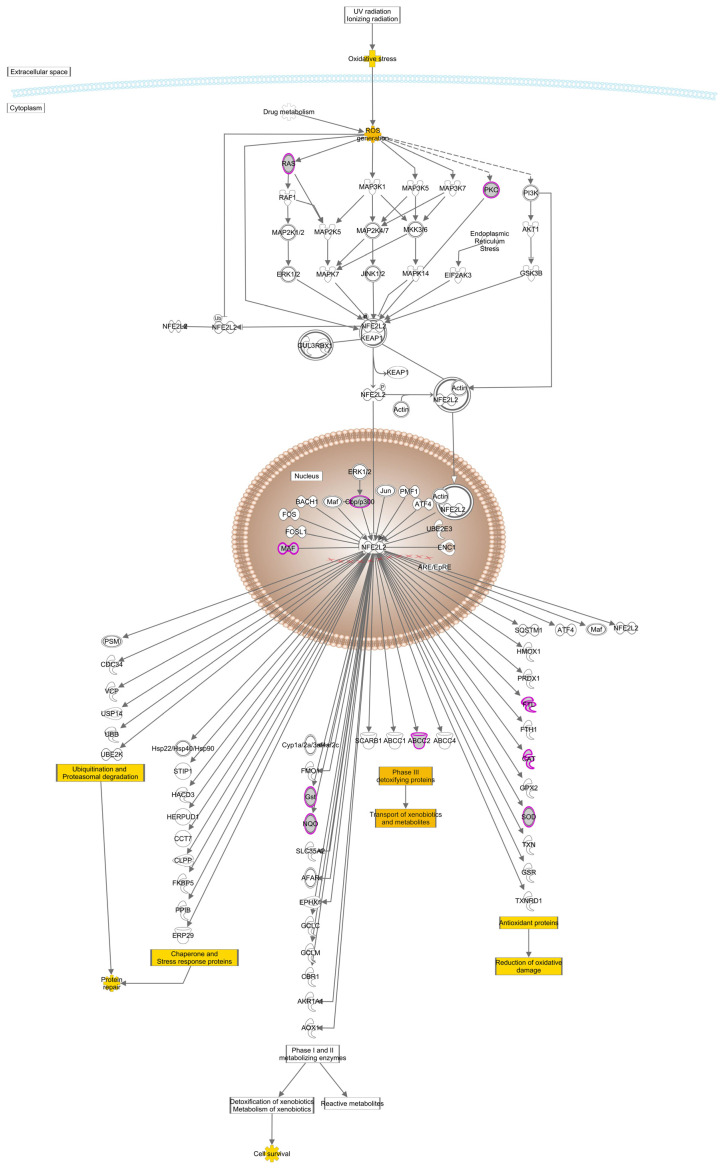
Schematic diagram of the NRF2 (NFE2L2) mediated oxidative stress response pathway. Location within the cell is shown for cytoplasmic and nuclear proteins. Genes associated with age-related cataracts are shaded gray and stresses, outcomes, and downstream pathways are shaded yellow.

**Figure 6:**
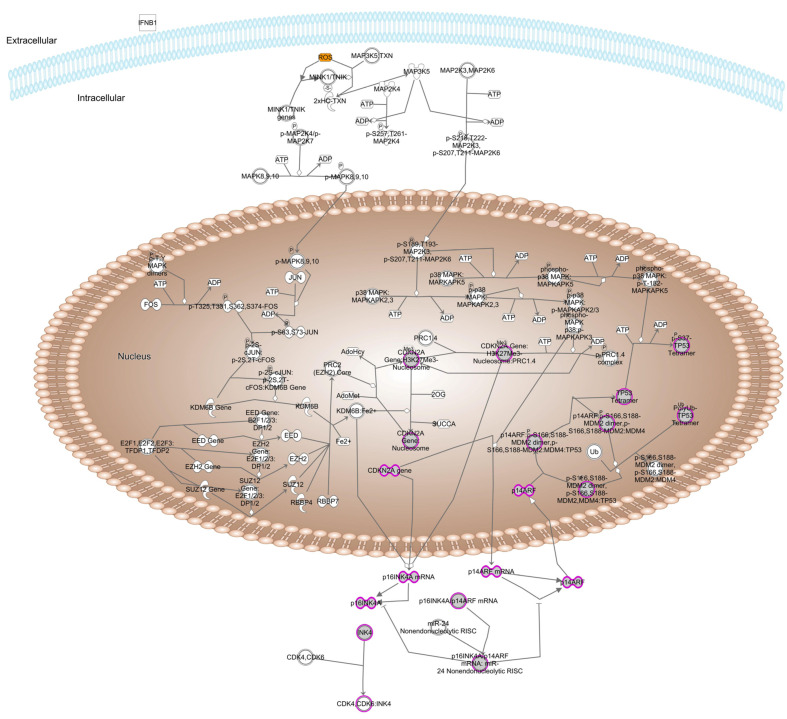
Schematic diagram of the oxidative stress induced senescence pathway. Location within the cell is shown for cytoplasmic and nuclear proteins. Genes associated with age-related cataracts are shaded gray and stresses are shaded yellow.

In addition to the effectors of antioxidant activity, a number of genes in transcriptional pathways regulating their expression have been implicated in age-related cataracts. The most enriched pathway in this regard is that of NFE like bZIP transcription factor 2 (NFE2L2, NRF2), (Figure 5). Oxidative stress and elevated ROS induce the small GTPase HRAS and protein kinase C δ, both of which are associated with age-related cataracts. These act through the MAPK/ERK and MAPK14 pathways, respectively, to dissociate NFE2L2 from KEAP1 [60]. NFE2L2 is then phosphorylated and translocates into the nucleus, where it forms heterodimers with MAF, also associated with age-related cataracts [61,62]. NFE2L2 transcriptional regulation is also modified by CITED2 (Cbp/p300 interacting transactivator with Glu/Asp rich carboxy-terminal domain 2), also associated with age-related cataracts. The NFE2L2-MAF dimers bind to AREs in the regulatory regions of NFE2L2 target genes and stimulate expression of both antioxidant proteins including catalase, FTL, and superoxide dismutase, repair enzymes including the glutathione-S transferases and NAD(P)H quinone dehydrogenase, which prevents single electron reduction of quinones that produces free radicals, and the multispecific transporter ABCC2 [62,63,64]. All of these have been associated with age-related cataracts. Conversely, once a combination of environmental stresses acting against a background of genetic susceptibility have resulted in sufficient oxidative stress, CKDN2A is activated, leading to increases in both of its splice products, p16INK4 and p14ARF (Figure 6), the latter of which stabilizes TP53 [65], which not only can induce apoptosis, but is directly associated with age-related cataracts [66]. The C allele of rs78378222, located in the 3′-untranslated region of the age-related cataract associated gene TP53, was shown to create a exact 8 bp binding site for miR-125b. In cultured lens epithelial cells binding of miR-125b was shown to decrease TP53 expression, and this was shown to occur in lens epithelial cells from patients with age-related cataracts more than unaffected controls. In addition to being a direct link to apoptosis and cell death through TP53, this pathway forms a positive feedback loop once oxidative stress has become sufficiently severe.

## 5. Discussion and Overview

This brief review summarizes the role that genes involved in antioxidative pathways and especially in minimizing oxidative damage directly play in susceptibility to age related cataracts. While some of these genes are also implicated in congenital cataracts, their role in congenital cataracts probably relates to mutations with a severe effect on protein structure, stability, or function, while they probably contribute to age-related cataract through mutations that have a relatively small effect on the protein structure or function, or affect the expression of the gene [1]. These latter variations then increase susceptibility to environmental insults associated with age-related cataractogenesis, of which oxidative insults are preeminent. The role of mutations or variants in antioxidant proteins acting through increasing susceptibility to these oxidative insults is consistent with their being identified in age related cataracts, and it is not surprising that variants in antioxidant associated genes are associated with the risk of age-related rather than congenital cataracts [67].

Even mutations in genes that are themselves unrelated to antioxidants eventually result in oxidative stress, especially if they impact mitochondria [68]. This is demonstrated by the presence of mitochondrial dysfunction in the top few canonical pathways and citric acid cycle and respiratory electron transport further down the rankings (Figure 1, Tables S3 and S4). In addition, acylglycerol kinase and a number of mitochondrial mutations have been implicated in cataract (CAT-MAP). Perhaps for this reason, many antioxidant agents being developed to prevent or delay age-related cataracts target mitochondria [69,70,71,72,73]. This is also the case for hyperglycemia, which elevates superoxide in the mitochondria [74,75,76,77,78]. Thus, the pathogenic mechanisms of diabetic and hyperglycemic induced cataracts are also implicated in age-related cataracts, although the glucose metabolism pathway does not reach statistical significance (Tables S3 and S4). This being the case, many of the proposed therapeutic approaches to diabetic and hyperglycemic induced cataracts, also target oxidation, mitochondria or mitochondrial processes [79,80,81]. While these approaches have not proven themselves in human trials yet, they do provide hope that novel human therapies might become available in the future. However, preventative approaches are complicated by identifying those individuals who will require therapy before the actual opacity occurs.

A number of caveats apply to the results summarized here. The first is that, in contrast to the genes identified as causes of Mendelian congenital or childhood cataracts, which often have functional support for their causality, most of the genes identified through association studies have not yet undergone a rigorous demonstration of their biological relevance to cataractogenesis and are merely probabilistically associated with cataracts in study populations. This is problematic, even when corrections for multiple testing have been applied, and is exacerbated in studies using a small sample size and by selection of genes for more recent targeted studies being biased by previous reports of association, especially when combined with a bias for reporting positive associations [82]. In addition to problems of statistical uncertainty, association is dependent on the mutation and population history, so that varied and even apparently conflicting results can be obtained in studies of different populations [83]. As an example, various association studies have shown association with GSTM1, GSTP1, and GSTT1 in various combinations or a lack of association with any of them [52]. These difficulties are complicated by the lack of suitable animal models for a process that often takes decades to develop in humans, and also the lack of a cell culture system that recapitulates the biology of lens fiber cells, which do not divide and do not turn over their protein components.

Several of the genes suggested by association studies do have biochemical support for their contribution to age-related cataracts. This includes glutathione peroxidase, for which knock-out mice develop age-related cataracts and catalase, which contributes to degradation of hydrogen peroxide in the lens, even though knocking it out in a mouse model does not cause cataracts [57,58]. TP53, while not identified in the GWAS or TWAS studies, also has functional support for a role in age related cataracts [66]. In addition, CRYAA, which was also not identified in the antioxidant pathway analysis, showed association with age-related cataracts in two of the GWAS studies [4,6]. While it is not known to be regulated by NFE2L2 and has no binding sites for NFE2L2 in or near its promoter region, it would fit well into the group of chaperones and stress response proteins shown in the NFE2L2 pathway (Figure 5). The T allele of rs13053109 in the promoter region of CRYAA is associated with increased risk of age-related cataracts and shows increased binding by KLF10 with consequent decrease in CRYAA transcription [84], providing indirect support for the role of the NFE2L2 pathway in age-related cataractogenesis. 

## 6. Conclusions

Thus, while because of the limitations of the studies on which they are based the list of pathways and proteins identified by genetic studies is almost certainly incomplete, and the validity of some of them has not been verified functionally, they do provide strong support for the role of antioxidant proteins in age-related if not in congenital cataracts. In addition, they point to a number of antioxidant pathways, some of which are well appreciated but some of which provide new avenues for exploration. Additional genes and pathways are likely to be identified in future studies, both in genetic epidemiology and functional analysis. Overall, association of antioxidant related genes and pathways with age-related cataracts currently might best be considered as identifying these pathways and emphasizing their importance in the lens’ defense against oxidative damage and opacity over time. 

## Figures and Tables

**Table 1:** Oxidative stress-related genes included in either CAT-MAP or a GWAS or TWAS. The pathways listed are from the IPA analysis and the morphologies of the cataracts and any associated systemic diseases are taken from the references.

Symbol	Entrez Gene Name	Location	Mendelian Inheritance	Pathway	Cataract Type	Associations	Ref
CAT	catalase	Cytoplasm	None	detoxification of radical oxygen species, NRF2 pathways, superoxide radical degradation			[41]
CREBBP	CREB binding protein	Nucleus	None	NRF2 pathways	nuclear-lamellar	Rubenstein Tabi syndrome	[42]
FTL	ferritin light chain	Cytoplasm	AD	NRF2 pathways	nuclear, stellate, Y-sutural, crystalline		[43]
GPX1	glutathione peroxidase 1	Cytoplasm	AR	detoxification of radical oxygen species, glutathione redox reactions		hemolytic anemia	[44]
GSTM1	glutathione S-transferase mu 1	Cytoplasm	None	glutathione, glutathione redox reactions, NRF2 pathways	cortical, nuclear, mixed		[45]
GSTP1	glutathione S-transferase pi 1	Cytoplasm	None	detoxification of radical oxygen species, glutathione redox reactions, NRF2 pathways			[46]
GSTT1	glutathione S-transferase theta 1	Cytoplasm	None	glutathione, glutathione redox reactions, NRF2 pathways	cortical (dln)		[46]
HRAS	HRas proto-oncogene, GTPase	Plasma Membrane	AD	NRF2 pathways		Costello syndrome	[47]
MAF	MAF bZIP transcription factor	Nucleus	AD	NRF2 pathways	cortical pulverulent, posterior polar, nuclear, lamellar, cerulean	Aymé-Gripp Syndrome	[48]
SOD1	superoxide dismutase 1	Cytoplasm	None	detoxification of radical oxygen species, NRF2 pathways, superoxide radical degradation	cortical, mixed		[44]

## Data Availability

All data used in this study are included in the manuscript.

## Supplementary Materials

The following supporting information can be downloaded at: https://www.mdpi.com/article/10.3390/antiox13111315/s1, Table S1: genes associated with age-related cataracts in each of the GWAS and transcriptome papers included in this review; Table S2: summed list of the genes identified in each type of study. Genes identified in CAT-MAP are included with the GWAS category in the right two columns rather than separately because both are association studies and the CAT-MAP gene list also references many of the identified GWAS papers; Table S3: IPA canonical pathways identified with the GWAS + CAT-MAP gene list; Table S4: IPA canonical pathways identified with the combined GWAS + CAT-MAP + Transcriptomic gene list; Table S5: antioxidant pathways significantly associated with age-related cataract genes and the genes identified within each pathways. Table S6: Explanations of abbreviations and symbols used.
